# Environmental Scanning as a Public Health Tool: Kentucky’s Human Papillomavirus Vaccination Project

**DOI:** 10.5888/pcd13.160165

**Published:** 2016-08-18

**Authors:** Amanda Wilburn, Robin C. Vanderpool, Jennifer R. Knight

**Affiliations:** Author Affiliations: Amanda Wilburn, Jennifer R. Knight, University of Kentucky College of Public Health and University of Kentucky Markey Cancer Center, Lexington, Kentucky.

## Abstract

Borrowing from business, quality improvement programs, and strategic planning principles, environmental scanning is gaining popularity in public health practice and research and is advocated as an assessment and data collection tool by federal funding agencies and other health-related organizations. Applicable to a range of current and emerging health topics, environmental scans — through various methods — assess multiple facets of an issue by engaging stakeholders who can ask or answer research questions, exploring related policy, critiquing published and gray literature, collecting and analyzing qualitative and quantitative data in both primary and secondary forms, disseminating findings to internal and external stakeholders, and informing subsequent planning and decision making. To illustrate the environmental scanning process in a public health setting and showcase its value to practitioners in the field, we describe a federally funded environmental scan for a human papillomavirus vaccination project in Kentucky.

## Background

Environmental scanning is a process used by businesses and other organizations to assess internal strengths and challenges and external opportunities and threats. Decision makers use environmental scans to collect, organize, and analyze data on their assets and shortcomings in external and internal environments to guide strategic planning and decision making (1–3). In business, environmental scans focus on acquiring relevant and credible information through various methods, including literature reviews, online database assessments, social media scanning, policy reviews, competitor appraisal, and solicitation of stakeholders’ opinions (eg, customers, board, staff), among other strategies (3). When properly executed, this process leads to a series of evidence-based responses that an organization can use to improve strategy and performance (4).

Recently, environmental scans were used to collect, organize, and analyze information on issues and practices in public health and medicine to look for quality improvement opportunities and research priorities, guide interventions, educate decision makers, and improve health outcomes. Environmental scans were used to address a range of topics, including chronic disease self-management (5), cancer care (2,6–8), mental health (9–11), injury prevention (12), and quality improvement programs (13–16). Environmental scanning integrates multiple strategies for information collection (2,17,18), including focus groups, in-depth interviews, and surveys with patients and providers; literature assessments; medical chart reviews; personal communications; review of internal documents; and policy analyses.

Similarities and differences exist between environmental scans and traditional public health evaluation principles. For example, similar to the Centers for Disease Control and Prevention’s (CDC’s) Framework for Program Evaluation in Public Health, an environmental scan has standards of utility, feasibility, propriety, and accuracy; it also has standards for engaging stakeholders, describing a program, focusing program design, gathering evidence, and sharing results (19). Additionally, an environmental scan and CDC’s framework both emphasize using lessons learned to improve public health effectiveness and sharing those lessons with stakeholders. The difference between CDC’s framework and an environmental scan is in the purpose. The purpose of an environmental scan is to understand context; collect information; and identify resources, links, and gaps whereas CDC’s framework evaluates the merit, worth, or significance of a program or policy. When a program or policy is evaluated in CDC’s framework, evidence is gathered and conclusions are justified to judge performance and determine whether program goals and objectives were accomplished. In an environmental scan, activities focus on understanding the internal and external environment of a particular topic and providing input into strategic thinking, decision making, and planning (2,3).

Despite its adoption as an assessment tool in various health care contexts, an environmental scan does not have a consistent definition or process in public health practice. In some instances, an environmental scan is used as an informal catch-all term akin to a needs assessment (2); in other instances, it aligns with strategic planning and quality improvement initiatives (3,7,18,20). Additional application and critique of environmental scans is needed to improve the effectiveness of this tool and related methodology (5). In recognition of the utility of environmental scans in public health practice and the need for more applied examples, in this article we describe the steps for an environmental scan and use as an example the environmental scan that we conducted of a federally funded human papillomavirus (HPV) vaccination project in Kentucky. Our goal is to help public health practitioners successfully apply this methodology in the context of public health practice and research.

## 7 Steps of the Environmental Scan for Kentucky’s HPV Vaccination Project

In September 2014, eighteen cancer centers, including the University of Kentucky Markey Cancer Center, were awarded 1-year support from the National Cancer Institute (NCI) to conduct an environmental scan and collaborate with other organizations to increase HPV vaccination uptake in pediatric care settings (21,22). The scan’s design consisted of 7 steps that could be applied to many other public health areas. 

Elements of the environmental scan process were used by the Kentucky Cancer Consortium to address other public health issues, including exposure to secondhand smoke; barriers to colorectal cancer screening, obesity, and cancer; and the Affordable Care Act’s impact on cancer care (23). Lessons learned then contributed to creating and conducting our environmental scan for the HPV vaccination project. As we moved through phases of development, implementation, evaluation, and dissemination (Figure), we routinely shared our process and methodology with Kentucky Cancer Consortium’s membership and academic colleagues who have environmental scan experience to help inform our work (3). Following is an outline of the 7 steps we used to conduct our environmental scan; each step includes an illustration of how the step was implemented in Kentucky’s HPV vaccination project.

**Figure:**
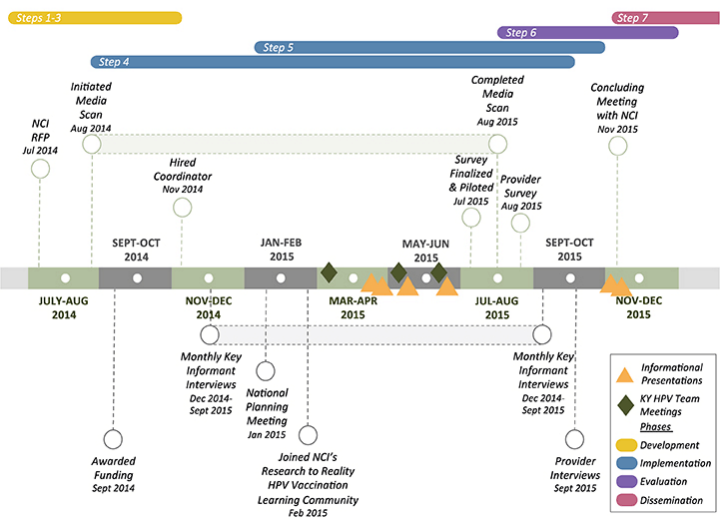
Timeline for developing and implementing an environmental scan for Kentucky’s human papillomavirus (HPV) project, July 2014–December 2015. Abbreviations: KY, Kentucky; NCI, National Cancer Institute; RFP, request for proposal.

### Step 1: Draw on experience to determine leadership and capacity for the project

A coordinator or team member must be designated to champion the entire environmental scan process from development to dissemination (3). Although resources vary by project and organization, an environmental scan must have dedicated leadership and clear roles and responsibilities for each team member. The scope and magnitude of the project needs to be within the organization’s capacity.

**Example from HPV vaccination project.** Two project leaders (R.C.V. and J.R.K) with complementary expertise in public health and cancer control drew on their experience in policy, systems, and environmental change; partnership development; community needs assessments; strategic planning; and health communication to develop the proposal for NCI funding and the overall environmental scan process. NCI required that one full-time coordinator (A.W.) be hired to conduct the HPV vaccination environmental scan; that coordinator would be responsible for day-to-day implementation.

### Step 2: Establish the focal area and purpose of the environmental scan

It is critical to specify a purpose for the environmental scan to anchor the process and focus the organization’s limited time, energy, and resources (3,20). Although the process can be flexible, a firm purpose will keep the environmental scan focused and its scope clear.

**Example from HPV vaccination project.** The purpose of the HPV environmental scan was to identify all public health activities, research, and information related to HPV vaccination in Kentucky, develop or improve links with existing programs, synthesize findings into a usable format for dissemination to stakeholders, and look for applied research opportunities to increase HPV vaccination uptake. The following definition was established by the investigative team:

A dynamic process of comprehensive assessment aimed at exploring HPV vaccination in a manner that makes connections not previously established and highlights barriers and facilitators not previously identified with the goal of empowering stakeholders with information for future strategic planning and decision making.

### Step 3: Create and adhere to a timeline and set incremental goals

Timelines may be imposed by a funding agency or, if not, by organizational leadership. If the environmental scan is independently organized (ie, not dictated by a funding agency), establish a timeline at the outset. Plan environmental scan activities to optimize the process and stay on task. For example, if surveys or qualitative interviews are part of the environmental scan, allocate appropriate time for creating the survey tool and interview guides, pilot testing the instruments, getting approval from institutional review boards, recruiting participants, collecting and analyzing data, and synthesizing and interpreting data.

**Example from HPV vaccination project.** Our 1-year timeline was set by NCI; having a timeline helped prioritize the scan’s components. Some components required attention during times specified by stakeholders; for example, the Kentucky HPV Initiatives Team met bimonthly and required us to schedule some activities accordingly. We planned to complete quantitative and qualitative data collection within 1 year (Figure). The time allocated for the provider survey, which was fielded in August 2015, included time for the following activities: developing the survey instrument through collaboration with other funded cancer centers; applying for university institutional review board approval; pilot testing survey constructs and preliminary questions with 6 clinicians; collecting responses from 231 physicians, midlevel clinicians, nurses, or pharmacists; and analyzing the preliminary data.

### Step 4: Determine information to be collected for the environmental scan

Brainstorm all topics and resources that could inform the environmental scan (2,3,7,18,20). All desired information may not be available, but include everything that, ideally, should be part of the scan. Casting a wide net and finding that information is unavailable is better than risking missing something important. Unlike Step 2, the list of items in this step will be dynamic, changing as opportunities to engage stakeholders develop and new resources are discovered.

**Example from HPV vaccination project.** The project started with several general areas related to HPV vaccination activities in Kentucky: state cancer registry and immunization data, media coverage, the policy environment, public health practice and research environments, a literature review, an update of the Kentucky Cancer Action Plan, other states’ HPV vaccination initiatives, 14 key informant interviews, and identification of research priorities. As the environmental scan progressed, several topics proved to be more robust than others. For example, the Kentucky Department for Public Health’s Division of Immunization received CDC funding to conduct a multimedia campaign promoting HPV vaccination during the back-to-school season. In other instances, staff had to seek unique sources. For example, the Kentucky Immunization Registry does not require that data on HPV vaccination be entered into its system; therefore, other data were obtained to help create a picture of HPV vaccination trends in Kentucky, including data from the CDC’s Comprehensive Clinical Assessment Software Application, a tool for assessing immunization coverage and practices in clinics and other places where immunizations are provided.

### Step 5: Identify and engage stakeholders

Stakeholders, and their willingness to participate in the environmental scan, are the key to success. Create a diverse, iterative list of people or organizations that have information on each topic named in Step 4. Stakeholders may expand the original list of topics by recommending or connecting project staff members to other stakeholders (ie, snowball approach).

Before approaching stakeholders, know what is needed from them. Create a plan for conversations with participants, whether it is a set of questions, requests, or action items. Be prepared to answer questions about the topic and environmental scan process as well as the funding requirements. Note all suggestions even if they do not seem pertinent at the time; they may prove valuable further into the project. Be prepared to offer something in return for their participation (eg, access to final environmental scan results or promotional materials).

**Example from HPV vaccination project.** During the NCI application process, we collected letters of support from local and state partners. These letters helped gain early support from established stakeholders. For example, the project coordinator had previously worked with the Kentucky Department for Public Health and had professional rapport with its immunization branch. In turn, the immunization branch told us of stakeholders unknown to the vaccination project team. The list of stakeholders quickly expanded to include local immunization coalitions, a practice-based pharmacy research network, and pediatricians in rural Appalachia who had success with HPV vaccination.

We gave stakeholders a brief introduction to the environmental scan and devised a plan to maximize efficient use of their time. Some stakeholders asked us to participate in their public health activities. For example, the Kentucky College Health Association asked the project coordinator to speak at its annual meeting about HPV vaccination. A minigrant through the University of Kentucky’s Appalachian Center allowed the team to incentivize (with $75 gift cards) the pediatricians identified as successful vaccinators to participate in qualitative, in-depth interviews; these 6 interviews were invaluable to the environmental scan. Another grant from the American Cancer Society allowed us to work with the University of Kentucky’s Center for the Advancement of Pharmacy Practice and a local pharmacy chain in Appalachian Kentucky to promote HPV vaccination outside the medical home.

### Step 6: Analyze and synthesize results from the environmental scan into a concise summary report

Analyze all collected data and triangulate the data according to the environmental scan plan (18,20,24). Document quantitative and qualitative results from survey instruments, key informant interviews, policy and media assessments, and literature reviews and synthesize the results into meaningful conclusions as they relate to the focus area (3). In addition, identify evidence-based research priorities or intervention target areas, and use the results to support decision-making steps and an action plan that will guide public health research or practice projects and that empowers partners to move forward.

**Example from HPV vaccination project.** Near the end of the funding period, staff began analyzing data from the provider survey; identifying common themes from the in-depth provider interviews; synthesizing information from the key informant interviews; and analyzing television program transcripts from the 1-year HPV vaccination media scan. All activities were conducted with the objectives of creating an HPV vaccination research agenda, identifying effective partnerships and policies for replication, and ascertaining priority educational and interventional areas for key stakeholders.

### Step 7: Disseminate results and conclusions to key stakeholders

Researchers and practitioners may arrive at the final product in several ways (3,18). For example, the funding agency may provide a template for summarizing data in a final report. If following such a template is not required or no such template exists, create one at the beginning of the project or at the end. In the report to stakeholders, address how well the initial, overarching question and its subtopics were answered and list informational sources. Make the results of the environmental scan available to the funding agency, the organization’s leadership, and those who participated in the process.

**Example from HPV vaccination project.** NCI did not provide a final reporting template for this project. The format was not determined at the start of the project; rather it took form around the informational sources established in Step 4 of the scan. We made the final report available in paper and poster presentation form for the funding agency, key stakeholders, and other interested parties. Additionally, the environmental scan team gave 6 informational presentations at national, state, and local conferences.

## Discussion

An environmental scan can be used to assess the external and internal environments of health programs or to identify barriers and facilitators to solving health problems in the context of a community or national priority area. An environmental scan may inform strategic planning and decision making for projects or interventions, guide the directions of a new public health activity, raise awareness of health disparities or other inequities, or initiate a project or funding opportunity (2,17,18). For example, HPV vaccination, although now recommended for more than a decade, is still relatively new on the public’s radar and is vastly underused in Kentucky and nationally for the prevention of HPV-related cancers (25–27). The environmental scan was a strategic and creative approach for NCI to gain a big-picture view of HPV vaccination activities in the catchment areas of 18 cancer centers. The 18 environmental scans provided NCI and each grantee with strategic, local information about links among cancer, immunization, and public health coalitions and programs to promote HPV vaccination; identified new collaborations aimed at increasing HPV vaccination uptake through applied research; and informed research and practice agendas, all with the goal of reducing the incidence of HPV-related disease.

Before starting an environmental scan, establish a working definition for an environmental scan (2). The definition needs to have detailed yet flexible steps to achieve the desired outcome, and the process must be fluid enough to allow for changes suggested by information gained from stakeholders and new questions that arise.

Perhaps the most important step of an environmental scan is to determine how to use the results (18,20). Share the final product (ie, hardcopy report, presentation) with stakeholders, including those who provided information for the environmental scan. Ideally, the final product will generate research priorities, identify funding gaps, create opportunities for effective intervention, and identify new partnerships for cultivation. Kentucky’s final report and poster presentation, made available to NCI and stakeholders, highlighted the need for robust HPV vaccination data, energized partners and identified new partners, and generated a list of research priorities, including conducting a pharmacy-based vaccination study and using community–clinical linkages to promote HPV vaccination.

Our description of an environmental scan has at least 2 limitations. First, a standard definition for or consistent approach to the environmental scan does not exist in the field of public health (2,18). The resulting ambiguity is a limitation of the process, and the definition and process will probably evolve as more public health organizations and practitioners adopt the tool. In time, the process described in this article may become more applicable or less applicable. Second, our environmental scan was conducted under one set of circumstances: it included funding and support from a federal agency, a full-time project coordinator, established relationships with key informants, and a 1-year timeline. The steps described in this article may not be generalizable to other public health environments. Regardless of these limitations, these environmental scan steps, or an adapted version of them, can be applied to many public health questions and areas of research and practice.
